# Copy Number Variation and Frequency of rs179008 in *TLR7* Gene Associated with Systemic Lupus Erythematosus in Two Mexican Populations

**DOI:** 10.1155/2022/2553901

**Published:** 2022-01-17

**Authors:** Guillermo Valencia Pacheco, Yumi E. Nakazawa Ueji, Julián Ramírez Bello, Rosa E. Barbosa Cobos, Eduardo D. Jiménez Becerra, Lizbeth J. González Herrera, Gerardo J. Pérez Mendoza, Nubia A. Rivero Cárdenas, Angélica V. Angulo Ramírez, Ricardo F. López Villanueva

**Affiliations:** ^1^Hematology Laboratory, Regional Research Center, Autonomous University of Yucatán, Yucatán, Mexico; ^2^Endocrinology Department, National Institute of Cardiology Ignacio Chávez, México City, Mexico; ^3^Rheumatology Department, Hospital Juárez de México, México City, Mexico; ^4^Genetic Laboratory, Regional Research Center, Autonomous University of Yucatán, Yucatán, Mexico; ^5^Rheumatology Department, Hospital General Dr. Agustín O'Horán, Health Service Yucatán, Yucatán, Mexico; ^6^Rheumatology Department, Regional Hospital General (ISSSTE), Health Service Yucatán, Yucatán, Mexico

## Abstract

Systemic Lupus Erythematosus (SLE) is an autoimmune disease in which genetic factors play a role in the susceptibility to develop it. Genes related to the synthesis of interferons such as *TLR7* and genetics factors such as single nucleotide polymorphisms (SNPs) or copies number variation (CNV) in the gene have been involved with the development of the disease. The genetic differences between the populations contribute to the complexity of LES. Mexico has a mestizo population with a genetic load of at least three origins: Amerindian, Caucasian, and African. The mestizo of Yucatán is the only group whose contribution Amerindian is mainly Mayan, geographically distant from other Mexican Amerindians. We analyzed the CNV and the frequency of SNP rs179008 of the *TLR7* as genetic risk factors in developing the disease in patients from Yucatán and Central Mexico. Results show that 14% of the cases of the Yucatecan population showed significantly >2 CNV and a higher risk of developing the disease (OR: 34.364), concerning 4% of those coming from Central Mexico (OR: 10.855). T allele and the A/T and T/T risk genotypes of rs179008 were more frequent in patients of Central Mexico than in those of Yucatán (50% vs. 30%, 93% vs. 30%, 4% vs. 1%), and association with susceptibility to develop SLE was observed (OR: 1.5 vs. 0.58, 9.54 vs. 0.66, 12 vs. 0.14). Data support the genetic differences between and within Mexican mestizo populations and the role of the *TLR7* in the pathogenesis of SLE.

## 1. Introduction

Systemic Lupus Erythematosus (SLE) is a chronic inflammatory autoimmune disease of unknown etiology, characterized by hyperactivity of B lymphocytes and the presence of anti-DNA autoantibodies, which affects women of reproductive age. It is a disease of universal distribution whose incidence and prevalence vary among populations [1–6]. In Mexico, a prevalence of 0.09% has been reported in Mexico City and 0.07% in Yucatán [7, 8].

Genetics play a role in the susceptibility to develop SLE, and the number of candidate genes associated with SLE has increased with the analysis of the human genome [9–11]; some are involved in the recognition of nucleic acids and production of interferons (IFN) [12, 13] while others are participating in T and B-cell signaling pathways [14, 15].

Genetic and epigenetic factors as SNPs and CNV play an essential role in autoimmunity. CNV of genes such as *C4*, *FCGR3B*, *CCL3L1*, and *TLR7* has been reported as risk factors in the development of SLE, to both susceptibilities to and to severity, as well as to other autoimmune diseases. CNV arises when a complete gene or segment of it is duplicated, having more than two copies, or when is deleted. Additional copies of these genes can promote overexpression of proteins, and its deletion leads to deficiency and functional changes [16].

Kelley et al. studied Caucasian and African American SLE patients to identify an increase in the CNV of the *TLR7* and its influence on the autoantibody profile. Their results indicate that the copy number of *TLR7* was variable between patients and controls, and no correlation with the phenotype of the disease, ethnic groups, and presence or absence of autoantibodies was found [17].

Garcia-Ortiz et al. investigated whether the CNV of *TLR7* contributes to the development of the disease in 328 Mexican pediatric patients and 403 controls. Their results showed a significant increase in the CNV in female patients compared to controls and a higher association in males (OR 6.61) than in female patients (OR 3.07). Their data indicate that the increase in CNV of *TLR7* may be a risk factor for developing the disease. Since *TLR7* is on chromosome X and there is an increased prevalence in women, data provides evidence of an X-linked genetic component in the susceptibility and pathogenesis of SLE [18].

Approximately 267 SNPs have been found in *TLR7* (GenBank Home) [19], and some are associated with the development of SLE. The SNP rs179008 (A>T) is in exon 3 of the *TLR7* gene, involves the exchange of a glutamine (Gln) for a leucine (Leu) at position 11 of the amino acid sequence, and it is related to the ability of TLR7 to recognize the uracil sequences located in the single-strand RNAs (ssRNAs), thus favoring high production of IFN-*α*. Gender differences based on *TLR7* may influence the SLE phenotype since women have higher levels of IFN-*α* compared to men. This data suggests that increased expression of TLR7, together with increased levels of IFN-*α*, contributes to the pathogenesis of SLE [20]. However, rs179008 has not been studied in the Mexican populations.

Considering the prevalence of SLE in the Mexican female population and the genetic heterogeneity among the mestizo subpopulations of Mexico, our objective was to determine the CNV and frequency of rs179008 of the *TLR7* gene as genetic risk factors to develop the disease in women with SLE from Yucatán where the Amerindian contribution is mainly of Mayan ancestry [21] and from Central Mexico with different ethnical groups.

## 2. Material and Methods

### 2.1. Selection of Study Populations

An observational and cross-sectional study was carried out using genetic material (DNA) stored at -20° C. Considering the calculations, we selected 100 samples of SLE patients and 102 healthy volunteers, all of them women of Mayan ancestry, with the inclusion criteria as individuals born in the country having a Spanish-derived last name, with Mexican Mayan ancestors back at least to the third generation, and at least one parent was born in Yucatán for two generations including their own. Women with Mayan ethnicity were selected using anthropological and demographic parameters such as language, place of birth, surnames, genealogy, and history of lifestyle to match ethnically all cases and controls. We also analyzed the samples of 151 SLE patients and 121 healthy volunteers, all of them women from the Central States of the country including Mexico City, belonging to different ethnical groups, whose samples were provided by the Department of Endocrinology, National Institute of Cardiology Ignacio Chavez, Mexico City. Patients diagnosed by a Rheumatologist, according to the criteria established by the ACR [22], signed the informed consent letter to participate in the study. Controls did not show any autoimmune or infectious disease upon entering the study and signed the informed consent letter. Pregnant women, and with other autoimmune diseases such as Rheumatoid Arthritis and Sjogren, were excluded. The Ethics Committee of Agustin O'Horán Hospital and Hospital Juárez de Mexico approved the study. The confidentiality of participants was strictly maintained.

### 2.2. Determination of CNV of the *TLR7*

DNA samples from patients and controls were quantified using Nanodrop equipment (260 and 280 nm) to verify their concentration and purity. CNV was determined by real-time PCR using TaqMan probes marked with FAM (Applied Biosystems Hs00226289_cn). Amplification was carried out in the StepOne real-time PCR ThermoCycler (Applied Biosystems®) using the TaqMan Universal Master Mix II reaction mixture. Thermal cycling conditions consisted of initial denaturation at 95°C for 10 minutes, followed by 40 cycles at 95°C for 15 seconds each and 60°C for one minute each. Three wells without genetic material and three with known DNA presented 2 CNV of TLR7 were included as negative controls to validate the assays.

CNV was estimated by the 2-^∧∧Ct^ method [23], which calculates the difference in cycle thresholds (the number of PCR cycles required to produce a set of fixed thresholds) between the gene of interest and the housekeeping gene (*RNAase P* gene, ∧C^t^), using the copy-caller software version 2.0© (Applied Biosystems). Subsequent calculations normalize the *∧C*^*t*^ of each sample to a calibrator (DNA with 2 CNV of *TLR7*) that has assigned a relative expression value of 1.00 (*∧∧C*^*t*^). Assuming that the amount of PCR product doubles with each successive PCR cycle, calculating the 2–^∧∧Ct^ value will provide the relative amount of DNA initially available for amplification in each quantitative PCR run. Therefore, the 2–^∧∧Ct^ method reveals differences in the gene relative copy numbers between the samples tested. A range for each expression value was calculated based on the standard deviations of the *∧∧C*^*t*^ value, where 2-(^∧∧Ct + *s*^) is the lower limit and 2-(^∧∧Ct-*s*^) is the upper limit.

### 2.3. Determination of the SNP rs179008

Allelic and genotypic frequencies were determined by allelic discrimination assays with probes TaqMan (c_ 2259574_10). Amplification was carried out in the StepOne real-time PCR ThermoCycler (Applied Biosystems®) using the TaqMan Universal Master Mix II reaction mixture. Thermal cycling conditions consisted of initial denaturation at 95°C for 10 minutes, followed by 40 cycles at 95°C for 15 seconds each, at 60°C for one minute each, and 60°C for 30 seconds each.

### 2.4. Statistical Analysis

CNV between patients and controls from both populations was analyzed employing the Wilcoxon signed-rank test of the Graph Pad Prism software. The association of CNV with SLE was determined using the MedCalc© V19.1.7 software comparing patients and controls. To determine if the increase in the CNV is associated with the disease, patients with >2 CNV of Yucatán and Central Mexico were compared, as well as patients with >2 CNV and ≤2 CNV with the total controls of both populations, using the EpiTools software.

Allelic and genotypic frequencies of rs179008 were calculated with the SNPStat© software (https://www.snpstats.net/start.htm). The association analysis was determined by comparing the genotypic and allelic frequencies between patients and controls using the MedCalc© V19.1.7 software. The risk of susceptibility to SLE, in terms of odds ratio (OR) and confidence interval (CI 95%), was used to determine whether alleles and genotypes of rs179008 represent a risk factor, considering as a reference value equal to 1. This data is null since it reflects a reason 1 : 1 between exposed and unexposed individuals. OR > 1 is considered as a risk factor, and <1 is protective. Values of *p* < 0.05 were obtained from two-tailed tests with the statistical package STATA 11.1.

The statistical power for a case-control study applicable to population genetics was estimated with the Quanto software (http://biostats.usc.edu/Quanto.html), using the frequency of 17% of the minor allele T of rs179008, reported in the 1000 Genomes Project Phase 3 (https://useast.ensembl.org/Homo_sapiens/Variation/Population?db=core;r=X:12885040-12886040;v=rs179008;vdb=variation;vf=140870338), prevalence of SLE in Mexico [7, 8], the dominant inheritance model, the hypothesis of a single gene hypothesis, and an OR = 2.5. This calculation yielded the sample size of cases and controls of both populations to achieve 80% statistical power.

## 3. Results

### 3.1. CNV Analysis of *TLR7* Gene

This is the first study comparing women with SLE from two Mexican populations; the Yucatecan from various municipalities of the Yucatán State with an essential contribution of Mayan ancestry, and from Central Mexico coming from the different Central States of the country (Mexico State, Guerrero, Queretaro, Oaxaca, Tlaxcala), which represent the ethnic heterogeneity in the last population.

**Figure 1:**
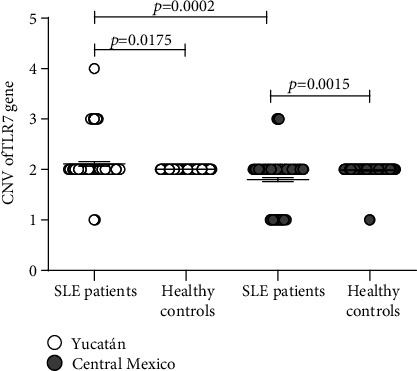
Distribution of CNV of the *TLR7* between SLE patients and healthy controls from Yucatán and Central Mexico. Differences in CNV between patients and controls were analyzed as described in Material and Methods. *p* values < 0.05 were considered significant.

Significant differences were found in the CNV between patients and controls and among patients, from both populations (Figure 1). We observed that 14% of Yucatecan patients showed significantly >2 CNV compared to 4% of those in Central Mexico and 34.36 times more at risk for developing the disease (Table 1). Patients with >2 CNV of Yucatán and Central Mexico were compared to determine if the increase in the CNV of the *TLR7* is associated with the disease; a significant association and 3.93 times more risk of developing the disease was observed in Yucatecan. Patients with >2 CNV from both populations were compared with total controls, and association and risk for developing the disease were observed in them, respect at patients with ≤2 CNV. The data suggest that having more than two copies of *TLR7* is a risk factor for developing the disease in both populations, the Yucatecan being at higher risk.

### 3.2. Allelic and Genotypic Frequencies of rs179008

Wild allele A was more frequent in SLE patients and healthy controls of both populations, while the risk allele T was less frequent in Yucatecan. However, the highest association of the T allele was observed in patients from Central Mexico, while in Yucatecan seems to be a protective factor (Table 2).

On the other hand, the A/T and T/T risk genotypes showed higher frequency and association in SLE patients from Central Mexico. Additionally, allelic and genotypic frequencies between SLE patients and controls of both populations were compared, and a significant difference between them was observed supporting the genetic differences between Mexican populations (Table 3).

## 4. Discussion

The susceptibility for developing SLE results from the interaction of multiple genes and environmental factors; however, ethnicity plays a vital role in its development, and the Amerindian population is more susceptible to developing it. Our country has a mestizo population with a genetic load of Amerindian, Caucasian, and African [24–28].

Silva-Zolezzi et al. evaluated the genetic diversity and ancestry of 600 Mexican mestizos proceeding from Sonora, Zacatecas, Guanajuato, Guerrero, Veracruz, and Yucatán, and they observed genetic differences between and within Mexican mestizo populations. The subpopulation of Yucatán is the only mestizos with an Amerindian ancestral component, mainly Maya, and represents an ethnic group geographically distant from other Mexican Amerindian groups [29]. This characteristic could be influencing the development of SLE, compared to the population of Central Mexico, supporting the importance of studying the CNV of genes involved in the disease.

Kelley et al. observed differences in the CNV of the *TLR7* in patients and controls Caucasian and African Americans but no association as a genetic risk factor for the development of SLE, which contrast with our results [17]. On the contrary, our data seem to correlate with Garcia-Ortiz et al. whose observed association (OR = 3.07, *p* < 1.00*E* − 04) of CNV of the *TLR7* with the disease in the female child population of Central Mexico [18]. Their results also support what we previously reported: > 2 CNV of the *TLR7* in women of Mayan descent [30]. Data suggest that the CNV of the *TLR7* is a risk factor for developing the disease in Yucatecan and Central Mexico women. Gender stratification was not possible since all patients were female; however, it is crucial to consider the participation of male patients in subsequent studies. On the other hand, having more or less than two copies of the *TLR7* does not mean that the person develops or presents the disease since it depends on the combination of genetic, environmental, and hormonal factors to trigger it.

There are few association studies of rs179008 (Table 4). Sánchez et al. found a higher frequency of the risk allele (T) of rs179008 in SLE females from the Spanish Caucasian population but was not associated with the development of the disease [31]. dos Santos et al. analyzed the allelic and genotypic frequencies of rs179008 in Brazilian female patients and observed that it might be a susceptibility factor to develop the disease [32]. Enevold et al. found no association of these polymorphisms with SLE in patients from the Danish population [33]. Lee et al. conducted a meta-analysis of the relationship between 12 TLR polymorphisms and SLE susceptibility. The authors included 26 studies that involved 11,984 patients and 14,572 controls and observed an association of rs179008 and SLE in African but not in the Caucasian population [34].

This is the first report of rs179008 in two Mexican populations, and a significant association of the T allele, and A/T and T/T genotypes, was observed in Central Mexico women and suggests that it is a risk factor for them to develop the disease but not in Yucatecan women. These results may be influenced by genetic differences between Mexican populations, supporting the participation of the ancestral component. It is convenient to emphasize that the Yucatecan patients constitute a population of Mayan women, without ancestral substructure and history until the third generation in Yucatán, unlike those patients in Central Mexico [21]. Furthermore, we cannot exclude other SNPs of the *TLR7* that may be contributing to the development of SLE since the rs179008 is in a region of known CNV, and alleles may differ with the number of copies.

The sample size with sufficient statistical power is critical to accomplish the genetic risk variant association in human complex diseases such as SLE. The statistical power of 80% is to avoid false-negative associations and determine a cost-effective sample size in large-scale association studies. Analysis testing a single SNP requires 248 cases while screening 500,000 SNPs requires 1,206, based on the following criteria: an OR of 2.5% disease prevalence, 5% minor allele frequency, 1 : 1 case/control ratio, and 5% error rate in an allelic test [35]. Analyzing a single SNP under a dominant model and an OR of 2.5, the sample size of our study has enough power (> 80%) to detect the effect of the rs179008.

## 5. Conclusion

The results support the role of the *TLR7* in the pathogenesis of SLE in Mexican mestizo and suggest that extra copies of the gene may be a risk factor for developing the disease in the Mayan population. The presence of the T allele, and A/T and T/T genotypes of rs179008, associated with the disease in patients from Central Mexico, supports the genetic differences between and within Mexican mestizo populations with different ethnical backgrounds.

## Figures and Tables

**Table 1:** Association analysis of CNV of the *TLR7* in SLE patients and healthy controls from Yucatán and Central Mexico.

Location	CNV	SLE patients *n* (%)	Healthy controls *n* (%)	OR	95% IC	*p*
Yucatán	≤ 2	86 (86%)	102 (100%)	34.36	2.02-584.46	3.00 *E*-04
	> 2	14 (14%)	N/D			
Central Mexico	≤ 2	145 (96%)	121 (100%)	10.85	0.60-194.64	7.10 *E*-02
	> 2	6 (4%)	N/D			
Yucatán	> 2	14 (14%)	—	3.93	1.45-10.61	8.40 *E*-03
Central Mexico	> 2	6 (4%)				
Yucatán/Central Mexico	> 2	14 + 6 (14% + 4%)	102 + 121 (100% + 100%)	2.76	1.02-7.46	4.40 *E*-03
Yucatán/Central Mexico	≤ 2	86 + 145 (86% + 96%)	102 + 121 (100% + 100%)	0.70	0.48-1.02	6.61 *E*-02

N/D: no detected.

**Table 2:** Association analysis of the allelic and genotypic frequencies of rs179008 (A>T) in SLE patients and healthy controls from Yucatán and Central Mexico.

Location	Allele and genotype	SLE patients	HW	Healthy controls	HW	OR	95% IC	*p*
Yucatán	A	168 (84%)		154 (75%)		Reference		
						0.58	0.3577-0.9620	3.4 *E*-02
	T	32 (16%)		50 (25%)				
	A/A	69 (69%)	0.45	58 (57%)	1	Reference		
	A/T	30 (30%)		38 (37%)		0.66	0.3669-1.200	1.7 *E*-01
	T/T	1 (1%)		6 (6%)		0.14	0.0163-1.1974	7.3 *E*-02
Central Mexico	A	152 (50%)		148 (61%)		Reference		
						1.55	1.1025-2.1896	1.2 *E*-02
	T	150 (50%)		94 (39%)				
	A/A	5 (3%)	< 0.0001	30 (25%)	< 0.0001	Reference		
	A/T	140 (93%)		88 (73%)		9.54	3.5696-25.5252	< 1.0 *E*-04
	T/T	6 (4%)		3 (2%)		12	2.2400-64.2865	3.7 *E*-03

**Table 3:** Comparison of allelic and genotypic frequencies of the *rs179008* between SLE patients and healthy controls from Yucatán and Central México.

*rs179008*	SLE patients Yucatán vs. SLE patients Central México	Healthy controls Yucatán vs. healthy controls Central México
A/A A/T T/T	*p* = <1.00*E* − 07	*p* = 1.00*E* − 06
A T	*p* = <1.00*E* − 07	*p* = 1.20*E* − 03

**Table 4:** Studies on rs179008 of *TLR7* associated with SLE in Mexico and other populations.

	Population	SLE patients/control	OR (95% CI)	*p*	Ref.
rs179008	Yucatán/Central Mexico	251/223	1.53 (1.102-2.189)	1.20 *E*-02	^∗^
	Spanish Caucasian	752/1107	0.97 (0.80-1.54)	7.00 *E*-01	[31]
	Brazilian	370/415	1.74 (1.2-2.70)	3.00 *E*-03	[32]
	Danish	142/443	NA	US	[33]
	African	11,984/14,572	0.43 (0.238-0.775)	5.00 *E*-03	[34]

NA: no association reported; US: unreported significance. ^∗^Current study.

## Data Availability

The SNP rs179008 data of *TLR7* gene, as well as softwares used to support the findings of this study, are included within the article.
